# An Observation Capability Semantic-Associated Approach to the Selection of Remote Sensing Satellite Sensors: A Case Study of Flood Observations in the Jinsha River Basin

**DOI:** 10.3390/s18051649

**Published:** 2018-05-21

**Authors:** Chuli Hu, Jie Li, Xin Lin, Nengcheng Chen, Chao Yang

**Affiliations:** 1 Faculty of Information Engineering, China University of Geosciences (Wuhan), Wuhan 430074, China; huchl@cug.edu.cn (C.H.); lijie_gis@cug.edu.cn (J.L.); lx@cug.edu.cn (X.L.); 2 State Key Laboratory for Information Engineering in Surveying, Mapping and Remote Sensing, Wuhan University, Wuhan 430079, China; cnc@whu.edu.cn

**Keywords:** semantic sensor web, observation capability ontology, sensor selection, observation planning, flood satellite sensors, flood observation

## Abstract

Observation schedules depend upon the accurate understanding of a single sensor’s observation capability and the interrelated observation capability information on multiple sensors. The general ontologies for sensors and observations are abundant. However, few observation capability ontologies for satellite sensors are available, and no study has described the dynamic associations among the observation capabilities of multiple sensors used for integrated observational planning. This limitation results in a failure to realize effective sensor selection. This paper develops a sensor observation capability association (SOCA) ontology model that is resolved around the task-sensor-observation capability (TSOC) ontology pattern. The pattern is developed considering the stimulus-sensor-observation (SSO) ontology design pattern, which focuses on facilitating sensor selection for one observation task. The core aim of the SOCA ontology model is to achieve an observation capability semantic association. A prototype system called *SemOCAssociation* was developed, and an experiment was conducted for flood observations in the Jinsha River basin in China. The results of this experiment verified that the SOCA ontology based association method can help sensor planners intuitively and accurately make evidence-based sensor selection decisions for a given flood observation task, which facilitates efficient and effective observational planning for flood satellite sensors.

## 1. Introduction

Currently, more than 1700 operational remote sensing satellites equipped with various sensors deployed on Earth [1] can be used for Earth monitoring (e.g., floods and landslides), forming a set of integrated and dynamic sensor networks to provide spatial-temporal and thematic observation information. In this context, selecting the right remote sensing satellite sensors to efficiently and effectively accomplish a specific Earth observation task is a key process in remote sensing satellite sensor management [2,3].

Observation capability information [4,5] is an important index for understanding the observational nature of the sensor and for qualifying the performance of sensor observations, which lays the information basis for sensor selection [6]. The formulation of a rapid and accurate sensor observation schedule depends upon both an accurate understanding of a single sensor’s observation capability and the interrelated multisensory observation capability information [4].

### 1.1. Sensor Discovery and Selection

In the current decade, the sensor web is an emerging paradigm for integrating multiple sensors into a WWW-based infrastructure that enables the interoperable use of sensors [7]. The Sensor Web Enablement (SWE) initiative developed by the Open Geospatial Consortium (OGC) defines several standard specifications to achieve this vision, for which sensor discovery and selection [6] are two basic steps. The Catalogue Service for the Web (CSW) of OGC defines a standard interface for discovering geospatial sensor resources by querying their metadata [8]. One SWE specification—Sensor Model Language (SensorML) [9]—is a flexible carrier for describing sensor information used for sensor discovery that has been applied in many SWE projects, including the European Sensors Anywhere (SANY) project [10], the Namibia Sensor Web project [11] and the Sensor Web 2.0 project [12]. The Sensor Instance Registry (SIR) [13] catalogue service adopts the SensorML 1.0-based discovery profile [14] to restrict the basic information model, which can address the sensor web resource discovery issue [15]. Although SensorML 2.0 [9] notes that the restriction of generic information models by defining the application-specific metadata profiles of specialized sensors should be included in future work, to date, the SensorML 2.0-based metadata profile for the accurate and fine-grained selection of Earth observation (EO) sensors has not been further formulated. In addition, the OGC EO metadata profile [16] describes the EO products’ metadata for discovering EO products, while this profile extends the observations and measurements properties from the view of EO sensor-derived data or products but not the sensor itself. To overcome the problem of missing sensor observation capability metadata and to effectively manage a large number of heterogeneous sensors, the SensorML-based observation capability information representation model [5,17] has been established for the fine-grained discovery of remote sensing satellite sensors. However, the correlated discovery of multiple sensors cannot be guaranteed [18].

Considerable semantics-based sensor discovery research has been conducted [19]. In 2008, the semantic sensor web concept [20] was proposed, which highlights annotating sensor data with spatial, temporal, and thematic semantic metadata. This approach uses the current OGC and SWE specifications and attempts to extend them with semantic web technologies to provide enhanced descriptions to facilitate the discovery of sensors and sensor-derived data [21]. The W3C Semantic Sensor Network (SSN) Incubator Group [6] has taken further advantage of semantic web technology to develop SSN ontology libraries that contain sensors, measurements, observations and so on [22]. The Linked Sensor Data application [23] based on SSN ontology and linked to one of the linked open data cloud knowledge bases, GeoNames, enables users to find all the sensors near the location of the users selected from GeoNames [24]. Additionally, the earth observation ontologies and linked geospatial data can be combined as a virtual sensor observation to enable the discovery of new knowledge for the EO monitoring application [25,26]. In conclusion, extant studies have solved the problem of sensor observation application to some degree; for example, in a given observation application, sensor inquiries can identify the list of qualified sensors. However, the available approaches are unable to select these sensors or sensor combinations to perform the concrete measurement; that is, they cannot answer questions such as, “which sensor can be employed with another sensor to measure the required variable in a given observation time and space for the particular application?”

### 1.2. Sensor Ontology

Sensor ontology plays an important role in semantic sensor webs that aim to realize interactions with and access to sensor and sensor-derived data on the web. One of the most remarkable works in sensor ontology development is the SSN ontology [22] that covers key sensor concepts such as sensor capabilities, properties, observations and measurement processes [27], which can be viewed as a watershed of sensor ontology development.

Prior to SSN ontology, several sensor ontologies had been established. Compton et al. [28] and Wang et al.[29] reviewed the typical sensor ontologies such as the CSIRO sensor ontology [30], which describes sensors, observations and scientific models for use in workflows, the CESN ontology [31], which considers a coastal environmental sensor with instrument, deployment, location and physical properties for inferring domain knowledge from coastal observation data, the A3ME ontology [32], which was developed to classify devices and their capabilities in a heterogeneous network, with a focus on making the ontology usable on resource constrained devices, the SWAMO ontology [33], which describes physical devices, process models and tasks for intelligent software agents, the MMI ontology [34], which is intended for marine equipment interoperability and focuses on system structure and observational processes and results, the OntoSensor ontology [35], which was created to build a knowledge base of sensors for querying and reasoning by setting sensors, capabilities descriptions and measurements as key framework concepts. These ontologies were built for their specific scope of application and have no universality.

The SSN ontology is resolved around the stimulus-sensor-observation (SSO) pattern [36], which follows the principle of minimal ontological commitments to develop a minimal set of classes and relations regarding stimuli, sensors and observations. Because SSN ontology has made great progress toward integrating and upgrading the original ontologies [22], its generality and broad extension space have enabled its extension and application to more complex problems [28,29]. Many applications extending from SSN ontology have appeared in recent years [37,38]. The IoT.est project [39] reused the SSN ontology in the internet of things to describe the resources, observations and measurement systems of sensors. The Semantic-Perception application [40] built a perception ontology based on the SSN ontology that can systematically derive abstractions from observations. The SECURE project [41] describes observational data from temperature sensors, carbon monoxide and dioxide sensors and can integrate and analyze the data and predict the environmental events. StarFL [42] is based on SensorML and SSN ontology; it defines a restricted vocabulary and model for sensor metadata. However, StarFL only demonstrates measurement capability (accuracy, resolution and range) when applied to generic sensor cases. The SemSorGrid4Env [43] project applies the SSN ontology to environmental monitoring systems. SWROAO ontology [44] serves as a knowledge repository of sensor web resources for the research and applications community in atmospheric science by describing the platforms, sensors and observations from aircraft, spacecraft and ground application perspectives. SCO ontology [45] represents new and major progress for the SSN ontology extensions, adding component, service and context modules. Recently, the new SSN ontology has been updated by W3C and OGC [46], which includes a lightweight but self-contained core ontology called Sensor, Observation, Sample, and Actuator (SOSA) for its elementary classes and properties. Although the new SSN ontology describes sensors and their observations, the involved procedures, the studied features of interest, the samples, the observed properties, and the actuators, the sensor observation capability ontologies have not been supplemented. In summary, existing sensor ontologies are mostly devoted to the description of basic physical information such as basic sensor features, observation data and physical observation properties. They all lack ontologies centered on sensor observation capability in detail.

### 1.3. Our Consideration

To accurately represent sensor observation capability and efficiently select multiple sensors, the following features are considered in the proposed sensor observation capability association (SOCA) ontology model:

(1) Covering the multilevel and multidimensional observation capability properties of the sensors. As defined in the Observations and Measurements (O&M) specification [47], the term “Observation” can consist of different observation types, including “Measurement” where the observation result is a numeric quantity, “CategoryObservation” where the observation result is a scoped name, “CountObservation” where the observation result is an integer number, “TruthObservation” where the observation result is a Boolean value, “TemporalObservation” where the observation result is a time-related object such as time instant, time period and date, “GeometryObservation” where the observation result is a space-related object such as the coordinate values of trajectory, position and location, and “ComplexObservation” where the observation result can be a more complex structure. In particular, this analogy yields the insight that sensor observation capability ontology should include the “MeasurementCapability,” “Category-ObservationCapability,” “Count-ObservationCapability,” “Truth-Observation Capability,” “Temporal-ObservationCapability,” “Geometry-ObservationCapability,” and “Complex-ObservationCapability” capability types.
(2) Supporting multi-sensor observation association. Providing only a compiled list of available sensors is far from sufficient. Decision makers often seek access to associated observation capability information among those sensors to facilitate the development of an efficient observation schedule (such as which sensors over what intersecting observation area can provide complementary observations of an observed phenomenon). Therefore, the proposed SOCA ontology that considers the dynamic associations among different sensor objects must be used as an information model for the correlated discovery of multiple sensors.

Table 1 summarizes the enablement of current sensor ontologies (Section 1.2) to support the features of the proposed SOCA ontology model. We conclude that (1) apart from the A3ME, SSN/new SSN, IoT.est and StarFL ontologies define the static measuring capability, the SWROAO and SCO ontologies partly describe the temporal-spatial observation capability, and few other observation capability ontologies are available, (2) a dynamic association among the observation capabilities of multi-sensors used for integrated observation planning is lacking, and (3) none of the ontologies can fully satisfy the requirements for accurately representing sensor observation capability and for efficiently selecting remote sensing satellite sensors.

Since SSN encourages domain experts to provide the domain-specific extensions to the SSN ontology, in this study, we propose a SOCA ontology model-based semantic association approach for the selection of remote sensing satellite sensors. This paper begins with the proposed sensor observation capability association (SOCA) ontology framework. Next, we analyze the key processes to realize the SOCA-based observation capability association and then conduct a flood satellite sensor selection experiment as a demonstration. The results and characteristics of the proposed observation capability semantic-associated approach are discussed. Finally, conclusions and directions for future work are presented.

The proposed model has the following novelties: (1) it performs as a descriptor to expand the description of sensor observation capability ontology and extends the association of multi-sensor observation capabilities, and (2) it acts as a dynamic and integrated observation capability information indicator that assists the decision maker in formulating a detailed and associated sensor observation solution for a specific observation task.

## 2. A SOCA Ontology for Sensor Selection

### 2.1. An Application Scenario

Suppose Mr. Min Li is an employee of the Changjiang Water Resources Commission (CWRC) in China and is monitoring a flood in the lower reaches of the Jinsha River. He usually identifies the appropriate sensors by retrievals from the China Centre for Resources Satellite Data and Application (CRESDA) or the Committee on Earth Observation Satellites (CEOS) databases. Rational sensor combinations are needed. Suppose that five qualified sensors have been identified from the global flood-supported satellite sensor resources. Before the observation capabilities of these sensors are correlated, the sensors are only discrete objects, and the observation capability association among them is unknown. After acquiring the integrated and dynamic observation capability association information in different dimensions, including temporal, spatial, thematic, and observation quality features, Mr. Li can select the sensors or sensor combinations in their rational observation mode on demand.

This example is used throughout the remainder of this article to clarify the role and use of the SOCA ontology. The SOCA ontology in this article can help Mr. Li to make a timely, evidence-based sensor selection decision for the specific flood monitoring task. The sensor selection solution, which was previously made manually by Mr. Li in a series of time-consuming checks among the information models of the available sensors, can be rapidly and accurately generated in an intuitive SOCA network environment.

### 2.2. Construction of SOCA Ontology

#### 2.2.1. Skeletal Methodology for Designing SOCA Ontology

From the ontological engineering perspective, establishing ontology in a standard process is necessary. The skeletal methodology is proposed by Mike Ushold and Michael Gruninger of the Department of Artificial Intelligence at the University of Edinburgh [48], which is the most classical methodology for building ontology, and can be used as a guideline for ontology development to include the following: identifying purpose and scope, ontology capture, ontology coding and evaluation.

**Figure 1:**
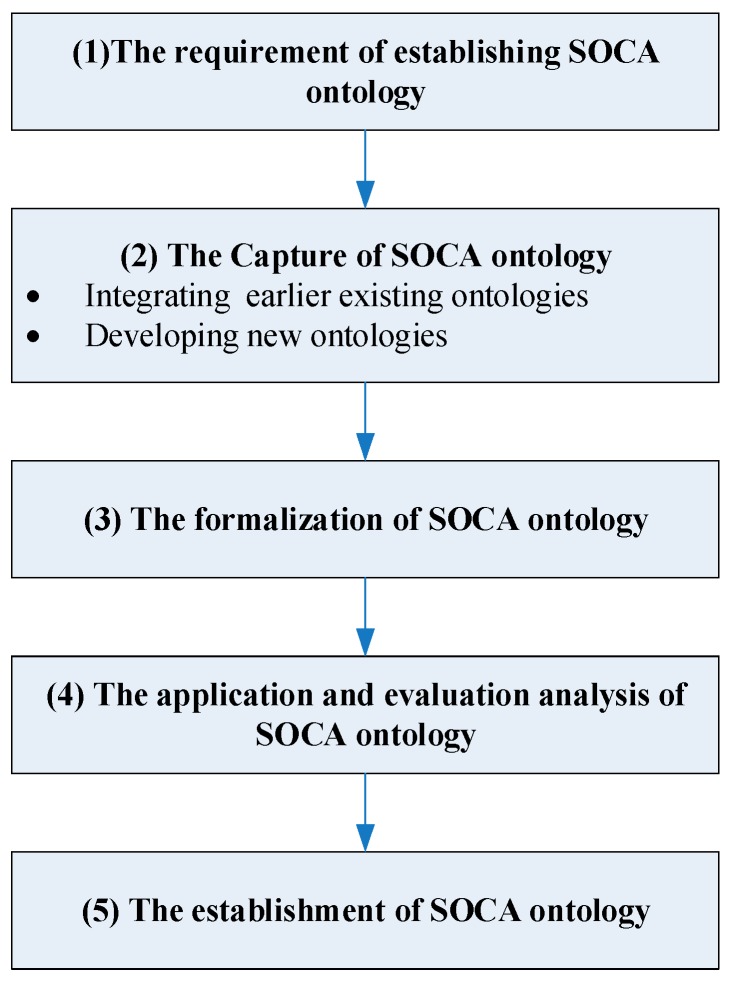
The skeletal methodology for designing SOCA ontology.

Figure 1 shows the SOCA ontology methodological architecture. On the basis of the steps of the skeletal methodology, this architecture comprises five stages:

- The requirement of establishing SOCA ontology: clarifying why the SOCA ontology is being built and what its intended uses are to help the ontology developers identify the purpose and the range of the SOCA ontology, which has been clarified in Section 1.3 and Section 2.1.
- The capture of SOCA ontology: identifying the ontology design pattern and modules, defining core classes and relations, reusing existing ontologies (e.g., SSN), and developing new ontologies to facilitate the generation of the complete SOCA ontology.
- The formalization of SOCA ontology: representing the conceptualization captured in the previous stage in some formal language (e.g., Web Ontology Language (OWL)) and ontology building tool (e.g., Protégé).
- The application and evaluation analysis of SOCA ontology: according to the intended application purpose to evaluate the efficiency, feasibility, usage, and extensibility of the SOCA ontology by some specified use cases.
- The establishment of SOCA ontology: starting the confirmation and publication of the well-evaluated SOCA ontology to assist the established ontology sharing and extensive application.

#### 2.2.2. SOCA Design Pattern

**Figure 2:**
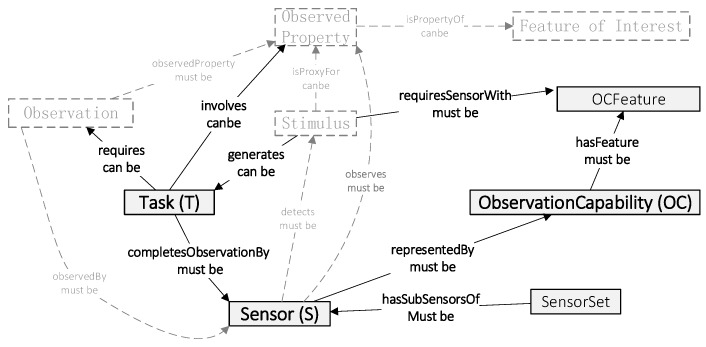
The core concepts and relations forming the Task-Sensor-ObservationCapability ontology design pattern considering and integrating the SSN ontology.

The SOCA ontology is centered on sensor observation capability concepts for the sensor selection domain in one particular observation task rather than a generic notion that includes observations, features, and properties. The task-sensor-observation capability (TSOC) ontology design pattern, which is presented in Figure 2, aims to accurately represent all kinds of sensor observation capability ontologies and vocabularies for the associated selection of multiple sensors in a certain observation task. The pattern follows the principle of minimal ontological commitments to make it extensible for a deeper-level observation application but focuses on facilitating sensor selection in one observation task. As shown in Figure 2, our TSOC ontology pattern is related to and expanded from the SSN ontology. The solid boxes and arrows are our proposed TSOC ontology. The dashed boxes and arrows represent the ontologies contained in the SSN ontology framework.

##### Stimuli

Stimuli [23] have been defined in SSN ontology as detectable changes in the physical environment that a sensor observes to infer information on environmental properties. They are the starting point of each measurement because they act as triggers for sensors.

##### Task

A task is an event that requires a manual or automatic observation response, e.g., a flood monitoring task requires a sensor manager to manually plan the available remote sensing satellite sensors for the related inundate flood water observations. Every task occurs in a specific time and space environment and has a unique observation property as well as personalized and dynamic observation requirements; for example, the resolution of spatial observations should be not less than 250 m, and the sky should be clear when the satellite passes. All types of tasks should find their qualified sensors for observation. The “Task” ontology defined here and the “Stimulus” ontology defined in SSN reflect changes in the environment; the difference between them is that the stimulus probably does not need to be observed. Take, for example, a rainfall event on Nanwang Mountain: because the rainfall is less than 3 mm/h, there is no possibility of landslides occurring in this event, i.e., this stimulus does not need a human observation response. The task thus serves as an adapter between stimulus and observation that will be triggered only if the change in the environmental phenomenon is greater than the threshold of a disaster. Therefore, every task needs an observation response.

##### Sensors

Sensors are physical entities. From the Earth observation perspective, sensors can be geographically applied in a series of activities, such as soil moisture, temperature and water flow monitoring. Each sensor has its own observation capability that can be used to observe a certain environmental phenomenon, e.g., sensor A can monitor changes in wind speed and direction with a digital output representation. After having been deployed in the Earth environment, these sensors can be used discretely or in an integrated way via sensor planning and controlling procedures.

##### Feature of Interest

Feature of Interest have been defined in the O&M standard [47] as entities in the real world that are the targets of sensing. In other words, Feature of Interest are the representations of the real world object the property is being estimated for. For example, water moisture, vegetation, buildings and other objects can be viewed as Feature of Interest of the physical environment.

##### Observed Property

As defined in the O&M standard [47], the Observed Property identifies or describes the phenomenon. It must be a property associated with the type of the feature of interest.

##### Observation

An observation is an act associated with a discrete time instant or period that results in an estimate of the value of an observed property of some feature of interest. It involves application of a specified procedure, such as a sensor, instrument, and algorithm or process chain. The procedure may be applied in situ, remotely, or ex-situ with respect to the sampling location [47].

##### Observation Capability

Observation capability is a collective concept that involves sensor information, such as “during which period”, “on which observation area”, “with what observed phenomenon” and “with what observation quality”, which can be used as the decision-making basis in one observation task for sensor planners to accurately utilize the sensors. Observation capability is used to understand and qualify the observation applicability of sensors. From the observation capability classification viewpoint, the static observation capability information is inherent in all sensors, while the dynamic observation capability information varies with the sensor random collocation and the real-time observation scene. Every sensor has its observation capability and can be represented by an observation capability information model [5]. The well-organized observation capability description can make the sensor well managed, i.e., fine-grained discovery of sensors and accurate selection of sensors has been enabled.

##### Observation Capability Feature

The observation capability feature involves concrete aspects or properties, which is the representation of the observation capability that should be contained in an observation capability set. The observation capability feature has two hierarchies around the sensor selection target: the static observation capability features such as spatial resolution and potential observation parameters are originally designed in the sensors, and the dynamic observation capability features such as the real-time observation coverage of a satellite sensor and the spatial observation relationship between two satellite sensors should be evaluated or calculated according to real circumstances.

##### SensorSet

Since the association among the different sensors in one observation application exists, a SensorSet is designed as a container to assemble those sensors and present their observation capability association records. For example, sensor i and sensor j are the flood observation-supported satellite sensors with the same observation resolutions; their images can be used to extract the flood area, and their ground resolutions are all better than 500 m; in this case, there are some observation capability association records that can be loaded in the SensorSet.

#### 2.2.3. SOCA Modules and Core Classes

**Figure 3:**
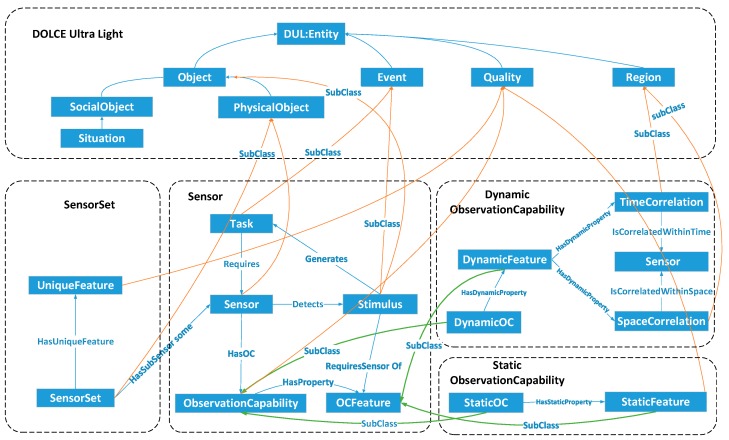
The SOCA ontology modules, core classes and relations aligned to DOLCE.

The SOCA ontology revolves around the central TSOC pattern (see the description in Section 2.2.2). Several conceptual modules build on the pattern to cover key sensor observation capability concepts. To assist knowledge engineers and users in interpreting the SOCA ontology, we align it to the DOLCE Ultra_Light (DUL) foundational ontology, which has been the top ontology of the engineering cognitive domain and in linguistics [49]. The DUL ontology defines 29 classes and 43 properties [50], including Entity, Event, Object, Quality Region, and Situation. Figure 3 shows the SOCA ontology modules, DUL-aligned core classes and relations, which will be detailed in the following sections.

##### Sensor Module

**Figure 4:**
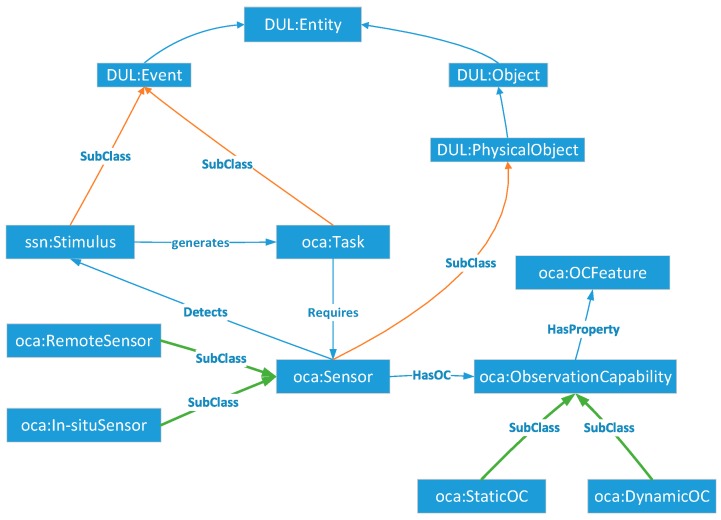
Core classes of the sensor module.

Sensors, in the SOCA ontology, as well as the sensors in the SSN ontology, are defined as subclasses of physical objects (*DUL:PhysicalObject*). As shown in Figure 4, *oca:Sensor* is related to *oca:Task* and *ssn:Stimulus* using the *Detects* and *Requires* relations, respectively.

Since the *oca:Task* and *ssn:Stimulus* are viewed as the representation of one particular event, the SOCA class *oca:Task* can be defined as a subclass of *DUL:Event*; the same applies to *ssn:Stimulus*. Sensors have observation capabilities for different subclasses such as remote sensing (*oca:RemoteSensor*) and in situ (*oca:In-situSensor*) sensors.

##### StaticOC Module

The StaticOC class is used to describe the static observation capability feature involved in sensor selection. Because *DUL:Quality* can represent the physical attributes of a specific physical object, *oca:StaticOC* can be a subclass of *DUL:Quality*.

**Figure 5:**
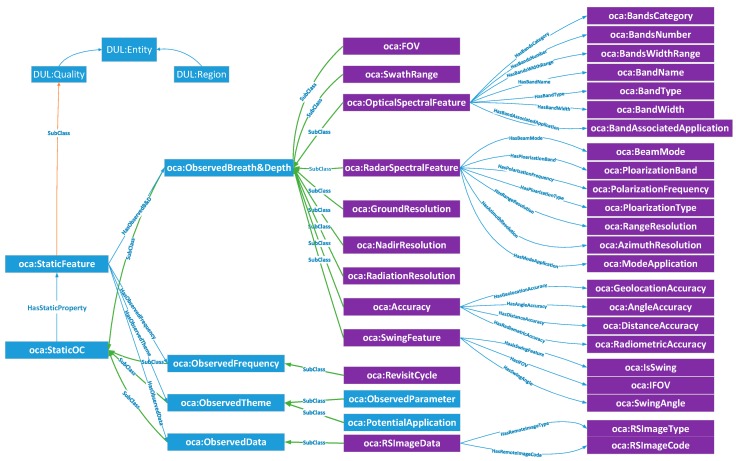
Core classes of remote sensing sensor StaticOC module.

This work focuses on the observation capability ontology of remote sensing sensors. Based on our established remote sensing sensors’ observation capability metadata model [5], four aspects in *oca:StaticOC* for remote sensing sensors have been further reformed (Figure 5): *oca:ObservedBreadth&Depth*, *oca:ObservedFrequency*, *oca:ObservedTheme*, and *oca:ObservedData*. *oca:ObservedBreadth&Depth* refers to the basic observation features derived from the horizontal and vertical scales of sensor observation capability, including *oca:FOV*, *oca:swingRange*, *oca:OpticalSpectralFeature*, *oca:RadarSpectralFeature*, *oca:GroundResolution*, *oca:NadirResolution*, *oca:RadiationResolution*, *oca:Accuracy* and so on. *oca:ObservedFrequency* is derived from the timescale dimension because evaluation of the time efficiency of sensor observations is important. *oca:ObservedFrequency* mainly includes the revisited observation cycle *oca:RevisitCycle*. *oca:ObservedTheme* presents the intended applications of the available satellite sensors, including *oca:observedParameter* and *oca:potentialApplication*. *oca:ObservedData* of remote sensing satellite sensors has subclasses of *oca:RSImageData* used to meet the specific requirements of sensor planners for observation results. 

##### DynamicOC Module

**Figure 6:**
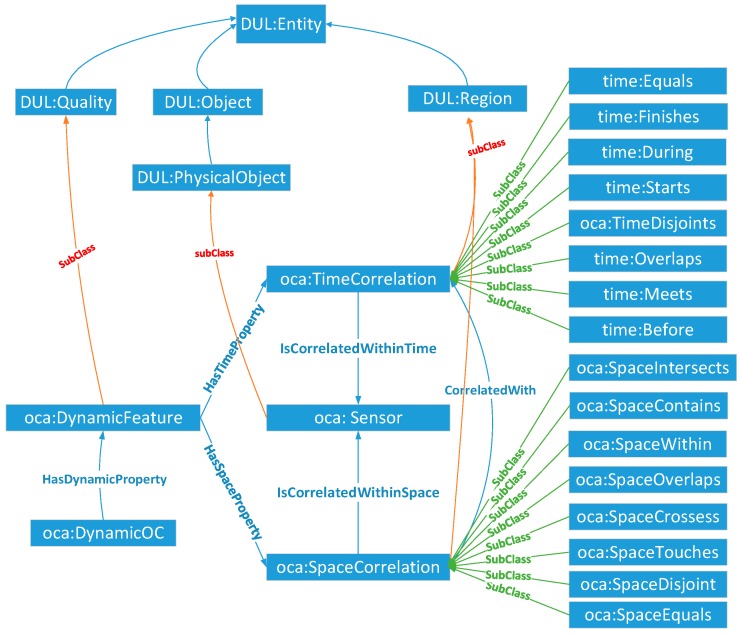
Core classes of the DynamicOC module.

The DynamicOC module of SOCA ontology is similar to the StaticOC module, which is a subclass of *DUL:Quality*. As shown in Figure 6, *oca:DynamicOC* refers to the dynamic spatial-temporal observation features according to the real observation scene. *oca:DynamicOC* uses *HasTimeProperty* and *HasSpaceProperty* relations and establishes two dimensional features: *oca:TimeCorrelation* and *oca:SpaceCorrelation*. The *oca:TimeCorrelation* and *oca:SpaceCorrelation* properties in the *oca:DynamicOC* class represent the spatial-temporal relations among different sensors and have different constituents. Because entities that have a *DUL:hasConstituent* property must be a *DUL:Region* [50], *oca:TimeCorrelation* and *oca:SpaceCorrelation* are the subclasses of *DUL:Region*. Following the nine-intersection model [51] of geographical information science for interpreting spatial topological semantics, and also referencing the existing spatial relationships (i.e., equals, disjoint, intersects, touches, crosses, overlaps, within, and contains) that are standardly defined in OGC simple feature access specification [52], *oca:SpaceCorrelation* reuses the existing spatial relationships including *oca:SpaceEquals*, *oca:SpaceDisjoint, oca:SpaceIntersects, oca:SpaceTouches, oca:SpaceCrosses, oca:SpaceOverlaps, oca:SpaceWithin,* and *oca:SpaceContains*. Similarly, the topological temporal relations of *oca:TimeCorrelation* can be reused from the current time ontology recommended by OGC/W3C [53], including *time:Equals*, *time:Finishes*, *time:During*, *time:Starts*, *time:Overlaps*, *time:Meets,* and *time:Before.*

##### SensorSet Module

**Figure 7:**
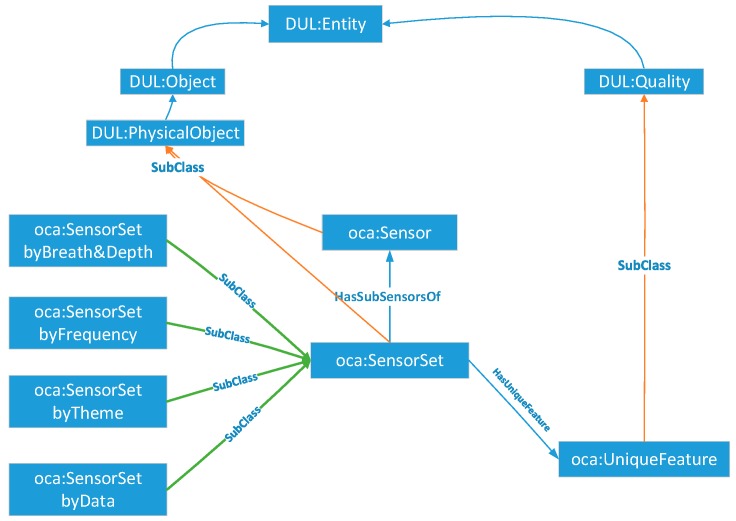
Core classes of the SensorSet module.

Since similarities exist among the static observation capabilities of different sensors, such as the spatial resolution of two sensors being better than 250 m and all having the water boundary extraction application, the SensorSet module performs as a package to carry the association relationships among those sensors. As shown in Figure 7, the core class *oca:SensorSet* is composed of a series of association sets (*oca:SensorSetbyBreadth&Depth*, *oca:SensorSetbyFrequency*, *oca:SensorSetbyTheme* and *oca:SensorSetbyData*) made by the comparison among one of the same dimensions of *oca:StaticOC* (*oca:ObservedBreadth&Depth*, *oca:ObservedFrequency*, *oca:ObservedTheme*, or *oca:ObservedData*) of the sensors. Each *oca:SensorSet* record has its *oca:UniqueFeature*, which is used to represent one unique association relationship among certain sensors.

#### 2.2.4. Formalization of SOCA Ontology

**Figure 8:**
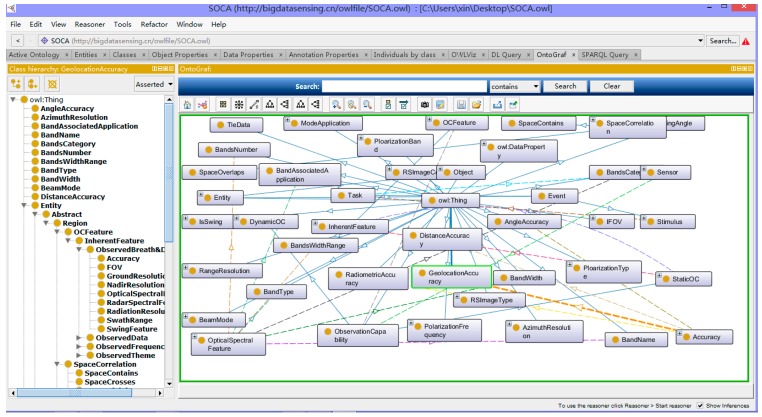
The graphical representation of sensor observation capability ontologies.

According to the ontology definition in Section 2.2.3, this section demonstrates the establishment of the SOCA ontology by using the Protégé ontology editor (Figure 8). Then the SOCA ontology in the OWL is generated, which can be viewed at http://bigdatasensing.cn/owlfile/SOCA.owl. In addition, the reviewer can on-demand check the ontologies according to the modules, classes and relationships from the website http://www.bigdatasensing.cn/SOCA.html.

## 3. SOCA Ontology-Based Observation Capability Semantic Association Implementation

### 3.1. Overall Framework

**Figure 9:**
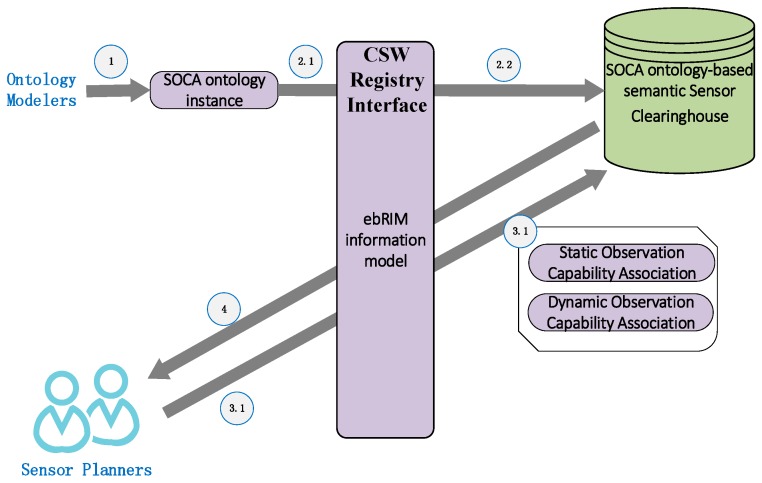
The overall flows to implement the SOCA ontology based observation capability semantic association.

As shown in Figure 9, four steps are involved to complete the observation capability semantic association. The first is to establish the SOCA ontology-based RDF instance. Ontology modelers can establish the remote sensing satellite sensors’ SOCA ontology-based RDF instance or convert SensorML-based remote sensing satellite sensors’ observation capability information model into a SOCA ontology-based RDF instance such as http://bigdatasensing.cn/RDFInstance/AVHRR3-NOAA.rdf. CSW is a representative and widely accepted registry service that has specific registry information models such as the ebXML Registry Information Model (ebRIM) [54]. In this research, the ebRIM-based CSW registry service was adopted. The second step is to convert the RDF-based SOCA ontology instance to the ebXML Registry Information Model (ebRIM); then, the ebRIM-based SOCA ontology instance can be directly stored into an ontology database such as the Oracle Database to form a semantic sensor clearinghouse. In this step, the related technologies, such as the mapping of the RDF-based ontology description to ebRIM, the semantic storage and registry, have been elaborated in our previous research, [55,56]. The third step is based on the GetRecords operation in the CSW specification; the sensor planners can enter a compound query on demand according to the observation requirements of the monitoring task. Additionally, the crucial process of the observation capability association is needed, which will be analyzed in detail below. After the processes of the Jena engine-adopted ontology reasoning and SPARQL-based querying, the last step is to return the multi-sensor association results to the sensor planners. 

### 3.2. Static Observation Capability Association

**Figure 10:**
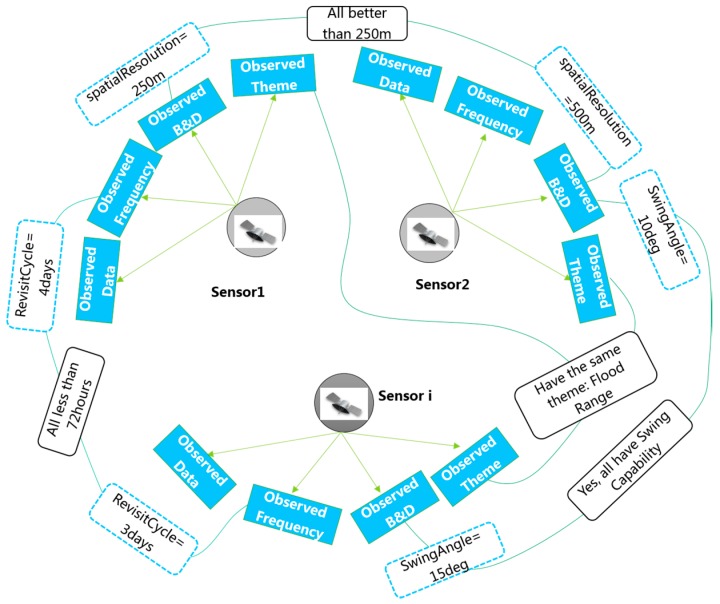
Example of static observation capability association among multi-sensors.

The association relationship among different sensors in the same dimension as their static observation capability can be established by combining similar items of the sensor static observation capability. Suppose that sensor 1 can observe the themes flood range, water level and water area with a spatial observation resolution of 250 m and that sensor 2 can observe the themes flood range and water pressure with a spatial observation resolution of 500 m. As shown in Figure 10, sensor 1 and sensor 2 can be merged in the *oca:observationTheme* dimension into the *oca:sensorSetbyTheme* container in which the *oca:uniqueFeature* of sensorSetbyParameter1 is flood range, which means that they all can be used for the flood range subject. Similarly, sensor 1 and sensor 2 can be merged in the *oca:ObservedBreadth&Depth* dimension into the *oca:sensorSetbyObservedB&D* container, and the association result is that their spatial observation resolutions are all better than 250 m. The other association relationships of the static observation capabilities between/among different sensors can be similarly constructed.

**Figure 11:**
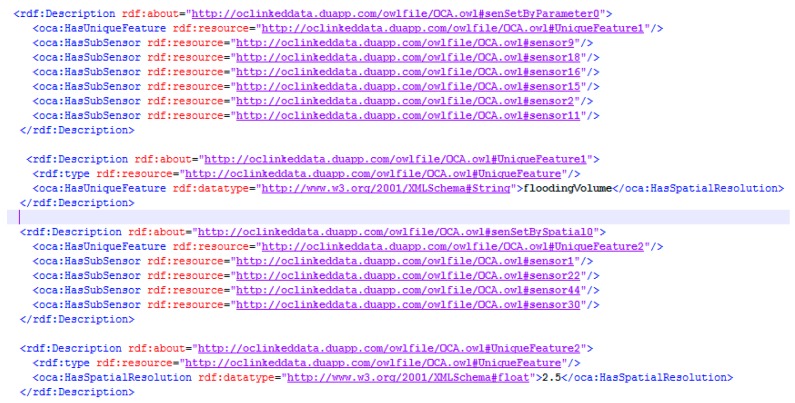
A part of an RDF-described static observation capability association instance.

Because of the advantages of the structural characteristics of the Resource Description Framework (RDF), we can formalize the static association results by the RDF, which also enables the establishment of semantic indexing. As expressed by the RDF in Figure 11, the association information (such as that sensor 9, sensor 18, sensor 16, sensor 15, sensor 2 and sensor 11 have the same observation parameter (flooding volume) and that all the spatial observation resolutions of sensor 1, sensor 22, sensor 44 and sensor 30 are better than 2.5 m) will become the semantic association information that can be understood by computers, thus greatly improving the efficiency and relevance of sensor searches oriented to one particular task.

### 3.3. Dynamic Observation Capability Association

**Figure 12:**
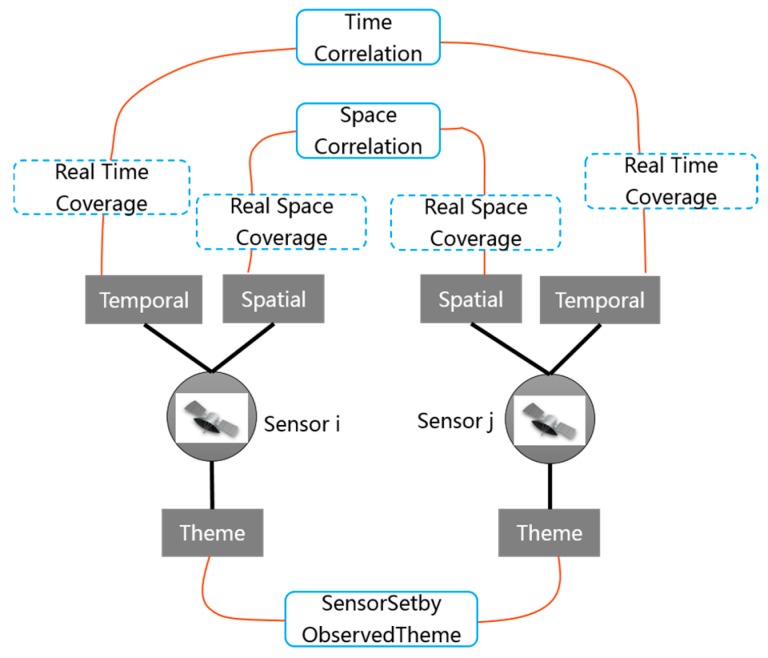
Sample of dynamic observation capability association among multi-sensors.

The combination method used to realize the dynamic observation capability association is similar to the static observation capability association. The difference is that the static observation capability association networks are established when the SOCA ontology instances are registered, whereas the dynamic observation capability association networks involving the spatial-temporal dynamic observation relations among different sensors can be established only under a specific observation task. The time correlations and spatial correlations can be referenced to the definitions in Section 2.2.3. Figure 12 illustrates the dynamic observation capability association network between two sensors in a specific task.

Similarly, the dynamic observation capability association information can be expressed by the RDF. An RDF-based observation capability association instance can be found in http://bigdatasensing.cn/OCAssociationSample.xml.

## 4. Sensor Selection Experiment for Flood Monitoring in the Jingsha River Basin

### 4.1. Overview of the Study Area

The Jinsha River basin is located in the upper reaches of the Yangtze River Basin and encompasses four provinces: Qinghai, Tibet, Sichuan and Yunnan. The total length of the Jinsha River basin, from Heyuan City in Qinghai Province to the Min River Estuary in Yibin City, Sichuan Province, is 3481 km, and the basin area is 502,000 km^2^, which accounts for approximately 26% of the Yangtze River Basin area. The terrain of Jinsha River basin is steep. The basin terrain of the western part is high, and that of the eastern part is low, forming a ladder-type distribution. The average annual flow of the Jinsha River is 4750 m^3^/s. Therefore, flood disasters in the middle and lower reaches of the Jinsha River have been caused by heavy snow melt in the upper reaches of the river, the inflow of many tributaries, heavy rainfall and rapid flow. Therefore, we selected the experimental area of the middle reaches of the Jinsha River basin as the study area.

### 4.2. Observation Query Inputs of the Flood Monitoring Task

*SemOCAssociation* is a prototype system developed by our team to provide the following functions: basic sensor discovery, multi-sensor association and association network visualization. According to satellite sensors statistics from the Observing Systems Capability Analysis and Review Tool (OSCART) of the World Meteorological Organization (WMO), 200 flood observation-supported satellite sensors were counted by our team for this experiment; the sensors can be checked at the website (http://www.bigdatasensing.cn/data/Flood_Observation-Supported_Satellite_Sensors.html). According to the static observation capability association process, more than one hundred thousand RDF-expressed association triples were constructed among the sensor static observation capability features.

Our *SemOCAssociation* prototype supports fine-grained flood observation queries, including basic spatial-temporal and parameter observation requirements and optional items such as spatial resolutions, time constraints, platform constraints, data constraints, sway angle constraints, and environmental constraints. Since flood monitoring is an extremely complicated process, different users will have different needs at different stages of floods. However, regardless of how complex the task requirements are, the time, space, and the parameters are the basic elements of the query criteria. Therefore, we set the initial query for this experimental flood observation task to the following: the observation time is (3 November 2017 10:00:00–3 November 2017 12:00:00); the spatial observation area is the middle reaches of the Jinsha River basin, and the geographic coordinate ranges are (UpperCorner: (N 29.6211°, E 99.7966°) (N 29.6211°, E 104.5616°)) and LowerCorner: (N 24.9905°, E 104.5616°) (N 24.9905°, E 99.7966°)); the fine-grained observation parameters are “flood range,“ “flow” and “water level”; the minimum spatial resolution is 500 m; and the other items are set as defaults with null values.

Using the basic sensor discovery function of *SemOCAssociation*, six satellite sensors were identified for the list of sensors qualified for the above observation query criteria: MVIRI_Meteosat-7 (interpretation tip: equipped sensor_satellite platform, similarly hereinafter), OP_CALIPSO, RA_HY-2A, ARGOS-4_NOAA-19, TIM_SORCE and VIRR_FY-3A.

### 4.3. Flood Satellite Sensor Selection

This section demonstrates the middle processes before the sensor selection decision, among which the SOCA ontology based static observation capability association and dynamic observation capability association are successively constructed. 

**Figure 13:**
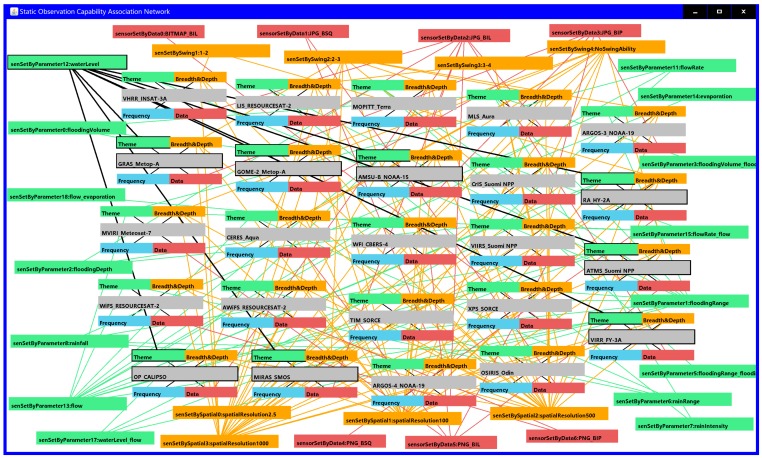
Example of the static observation capability association network for the qualified satellite sensors.

Figure 13 displays a part of the static observation capability association network composed of 24 satellite sensors (VHRR_INSAT-3A, LIS_RESOURCESAT-2, MOPITT_Terra, MLS_Aura, ARGOS-3_NOAA-19, GRAS_Metop-A, GOME-2_Metop-A, AMSU-B_NOAA-15, CrIS_Suomi NPP, RA_HY-2A, MVIRI_Meteosat-7, CERES_Aqua, WFI_CBERS-4, VIIRS_Suomi NPP, ATMS_Suomi NPP, WiFS_RESOURCESAT-2, AWiFS_RESOURCESAT-2, TIM_SORCE, XPS_SORCE, VIRR_FY-3A, OP_CALIPSO, MIRAS_SMOS, ARGOS-4_NOAA-19 and OSIRIS_Odin) that could be used to monitor flood range, flow or water level and have spatial resolutions better than 500 m. There were 800 RDF-expressed static observation capability association triples among these 24 sensors. Figure 13 shows a portion of the static observation capability association network, where different colors are used to represent the different association sets; for example, red represents the observation data association among sensors, orange is used to represent the observation breadth and depth association, and green represents the observation theme association.

**Figure 14:**
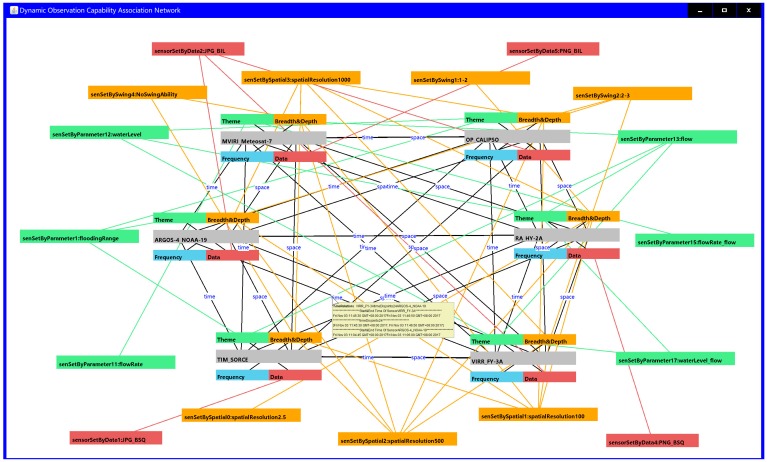
Example of the dynamic observation capability association network for the qualified satellite sensors.

In addition to matching sensors from the perspective of static observation capability, it was necessary to further filter the sensors that met the spatial-temporal observation requirements of the given flood monitoring task according to their dynamic spatial-temporal observation coverage situations. After two filter steps, six satellite sensors (MVIRI_Meteosat-7, OP_CALIPSO, RA_HY-2A, ARGOS-4_NOAA-19, TIM_SORCE and VIRR_FY-3A) were finally matched from the existing flood satellite sensor library. Compared with the static observation capability association, the dynamic observation capability association network increased the spatial-temporal association among sensors but decreased the sensors that did not satisfy the spatial-temporal observation requirements of the given task (Figure 14). A total of 75 RDF-expressed observation capability association records remained among these six sensors.

**Figure 15:**
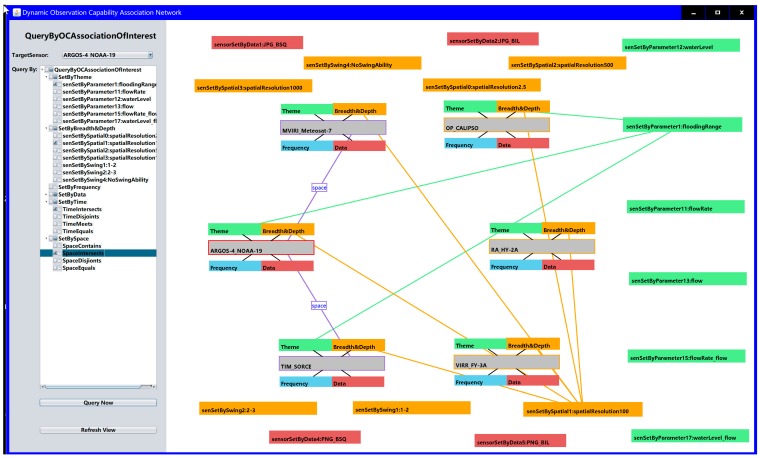
Supporting the retrieval of sensors and sensor associations of interest.

To efficiently and personally retrieve the sensors and sensor associations that sensor planners want to find from the vast, intricate observation capability association network, our prototype supports the mechanism of on-demand searching of the sensors and their associations by a series of combinational logic operations (Figure 15); i.e., sensor planners can select the target sensor and query which sensors can have the same observation parameters such as water level and form a spatial intersection with them.

## 5. Results and Discussion

### 5.1. Application Results of the SOCA Ontology

In this section, we present the effectiveness of the SOCA ontology which has been applied in remote sensing satellite sensors’ selection. First, we present the actual situation of multi-sensors’ association selection to compare whether the proposed SOCA ontology can be useful. We also evaluate the application effect of SOCA ontology in the *SemOCAssociation* system.

In the past, all sensors were organized in isolation in their observation capabilities’ representation [5]. That is, before the observation capabilities of these sensors are correlated, the sensors are only discrete objects, and the observation capability association among them is unknown. Therefore, the associated multi-sensor selection decision was made through a series of time-consuming checks among the discrete information models of those available sensors. For instance, sensor inquirers should compare each sensor information model and then laboriously conclude whether those compared sensors had the observation associations and determine what they were.

Now, when the sensor search criteria of the experiment are identified, the sensor inquirer can feasibly and easily find the sensors by semantic indexing based on the constructed RDF triples, which avoids the time-consuming comparison among the multiple sensor information models or the redundant retrieval from the full table of the observation capability database. The observation capability association networks are intuitively presented in Figure 13 and Figure 14. Sensor planners can thus achieve an intuitive understanding in the domain of the observing capability of sensors. Figure 13 shows the static observation capability association network of 24 sensors whose observation capabilities, except for the dynamic temporal and spatial observation conditions, met the requirements of the given flood observation experiment. There were 800 RDF triples in the static observation capability association network, where a sensor planner could explicitly read the correlation of these 24 sensors, such as OP_CALIPSO, MIRAS_SMOS, VIRR_FY-3A and other 5 satellite sensors, that have the same observation parameter water level, and LIS_RESOURCESAT-2 and MLS_Aura, which have swing capability and swing angles of between 3 and 4 degrees. Figure 14 presents the final observation capability association network after dynamic temporal and spatial observation filtration, in which six qualified satellite sensors were matched and 75 RDF triples were re-resolved. In this dynamic observation capability association network, sensor planners could accurately grasp the spatial-temporal observation association relationship between different two sensors (for example, TIM_SORCE forms a space intersection with MVIRI_Meteosat-7) and the coordinates of the intersected observation area are accurately indicated; the planners could thus understand the condition of whether TIM_SORCE and MVIRI_Meteosat-7 could be collaboratively selected for the observation theme of water level in a given observation time with a spatial observation resolution better than 250 m. Additionally, sensor planners could personally customize the target sensor and retrieve the association of interest such as the selection of ARGOS-4_NOAA-19 as the target sensor and select the association relationships “sensetByParameter1 = flooding range” and “sensetBySpaceCorrelation = space Intersects”. In this way, the given associations centered on ARGOS-4_NOAA-19 are retrieved on demand. In summary, the proposed SOCA ontology instance can effectively assist sensor planners in formulating a detailed and associated sensor observation solution when facing a complex observation task.

### 5.2. The Comparison between the Observation Capabilities in SOCA Ontology and the System Capabilities in the New SSN Ontology

The TSOC design pattern of our SOCA ontology takes a sensor planner-oriented viewpoint and emphasizes the semantics of the features of the sensor observation capability and their properties for the sensor selection application domain. This approach contrasts with the SOSA design pattern of the new SSN ontology, which takes a top-level and thus generally oriented viewpoint for understanding in the domain of sensing, sensor, sample and actuator, but do not emphasize the observation capability application. The O&M specification [47] defined that the term “observation” is used for the general concept. From the observation result type perspective, observation values may have many datatypes, including primitive types, such as measure or category, but also more complex types such as time, location and geometry. “Measurement” is reserved for cases where the result is a numeric quantity. As shown in Table 2, the new SSN ontology describes a set of measurement capabilities of a sensor in specific conditions. Compared with the SSN ontology, the SOCA ontology is a more comprehensive capability ontology that covers the multidimensional and multi-hierarchical observation-related capability feature. Moreover, it reflects the dynamic association among the spatial-temporal observation capability of multi-sensors. Specifically, apart from the measurement capability, CategoryObservation, CountObservation, TruthObservation, TemporalObservation, GeometryObservation, and ComplexObservation are supplemented in the SOCA ontology. Therefore, the SOCA ontology better interprets the nature of sensor observation capability.

Because the proposed SOCA ontology is aligned with DUL, its design framework can be reused by the other observation capability modeling requirements. Moreover, provided that the current maximum reusable observation capability ontologies may be unsuitable or unsatisfiable for certain individual requirements, our SOCA ontology model allows the capability modeler to expand or revise the observation capability fields in our current capability ontology model; that is, other new observation capability properties of a sensor can be represented as subclasses of the classes oca:BasicBreadth&Depth, oca:ObservedFrequency, oca:ObservedTheme, oca:ObservedData or oca:DynamicOC according to their meanings. However, the proposed observation capability ontology model framework is maintained.

### 5.3. Evaluation of SOCA Ontology Based on OntoQA Metrics

In most cases, the way where the ontology is built is largely dependent on the domain in which it is designed. Our SOCA ontology is used to enable the associated selection of multiple sensors in a certain observation task, which is different from the aim of other existing ontologies described in Table 1. Therefore, there are no “gold standard” measures of ontologies [57]. This section is to evaluate our SOCA ontology based on the OntoQA metric [58] which is a mainstream method to evaluate the quality of an ontology on the different dimensions.

In our constructed SOCA ontology, there are 75 classes (C) where the number of class-subclass relations (SC) is 37 and the number of non-class-subclass relations (nSC) is 38; there are 81 properties (P) where the number of object properties (OP) is 41 and the number of data properties (DP) is 40. Additionally, there are 200 SOCA ontology-based remote sensing satellite sensors’ RDF instances (I). Table 3 shows the self-evaluation of our SOCA ontology based on the OntoQA metrics, which can be used by ontology users or ontology developers to determine the internal quality of the proposed SOCA ontology.

### 5.4. Comparison with Other Satellite Sensor Management Platforms for Sensor Selection

As noted in Section 1, SIR and CSW are the existing web services for sensor discovery. In addition to the above sensor discovery services, open website-based search engines, such as Google, NASA’s GCMD retrieval portal (http://gcmd.nasa.gov/index.html), the CEOS satellite instruments catalogue (http://database.eohandbook.com/database/instrumenttable.aspx) and WMO OSCART (http://www.wmo-sat.info/oscar/instruments), are also available for discovering flood observation-supported satellite sensors. They are all characterized by the basic discovery methods that depend on keywords, structural catalogs, and plain text definitions for observable properties. For example, SIR allows a sensor planner to search for qualified sensors based on observed phenomena, observed data, temporal criteria, text fragments, units of measure, sensor ID and so on. CSW allows sensor inquiry limited by the search criteria such as sensor ID, static temporal criteria and spatial combination. Google facilitates sensor inquiries to determine suitable sensors using fuzzy modes such as the text fragments of the flood water surface emergency request. The GCMD portal refines the desired sensor resources according to the given free text. CEOS supports fine-grained directory query. The WMO OSCART database can be used for fuzzy queries based on text fragments. For all the above methods, the qualified sensor list can be matched, while those sensors are only association-unknowable resources. Precisely speaking, finding only the available sensor list does not mean that those sensors have been effectively managed. Therefore, compared with the above sensor discovery and planning systems, our *SemOCAssociation* system can induce sensor planners to generate sensor selection programs such as what group of sensors should be used, which sensor with what mode is to be combined with other sensors for what measurement parameters, and when do they start? All these advantages can be attributed to the proposed SOCA ontology based semantic observation capability association method.

## 6. Conclusions and Outlook

This study introduced a sensor observation capability association (SOCA) ontology model resolved around the task-sensor-observation capability (TSOC) design pattern. The pattern was developed considering the stimulus-sensor-observation (SSO) ontology design pattern, which focuses on facilitating sensor selection in one observation task. The core of the SOCA ontology model is to achieve the observation capability semantic association. The efficiency of the proposed SOCA ontology model was verified by applying it to sensor selection for flood observation in the Jinsha River basin. The results verified that the SOCA ontology based association method can help sensor planners make an evidence-based sensor selection decision for a given flood observation task, which facilitates the efficient and effective management of those flood observation-supported satellite sensors.

Current work mainly focuses on the qualitative effectiveness of the SOCA ontology for supporting the semantic-associated selection of multiple sensors. The quantitative efficiency of the SOCA ontology framework in judging the accuracy of an associated observation program will be evaluated in a follow-up study. Integrating our SOCA ontology instances into the linked open data cloud cannot be ignored in future work. In addition, because the current SOCA ontology considers only the observation capability of sensors themselves, the variation in observation capability with the required task has not been considered and will be added to facilitate the completeness of the SOCA ontology. 

## Figures and Tables

**Table 1:** Comparisons among current sensor ontologies.

	Features	Date	Target Object	Design Pattern	Application Usage	Supported Ontology Description	Supporting Multi-Sensor Association	Fine-Grained Observation Capability Description						
Sensor Ontologies								Measurement Capability	Category-Observation Capability	Count-Observation Capability	Truth-Observation Capability	Temporal-Observation Capability	Geometry-Observation Capability	Complex-Observation Capability
OntoSensor		2006	Generic sensor	N/A	Knowledge base and inference	Sensor physical property and observations	×	×	×	×	×	×	×	×
CESN		2008	Coastal Environmental Sensing Networks	N/A	Inferring domain knowledge from coastal data	Sensor types and deployments	×	×	×	×	×	×	×	×
A3ME		2008	Low-power devices	N/A	Devices and capabilities classification	Devices and their capability	×	○	×	×	×	×	×	×
SWAMO		2008	Intelligent software agents	N/A	Intelligent agents	physical equipment, the process model and tasks	×	×	×	×	×	×	×	×
CSIRO Sensor		2009	Generic sensor	N/A	Data integration, search, classification and workflows	Sensors and deployments	×	×	×	×	×	×	×	×
MMI		2009	Marine equipment	N/A	Marine equipment interoperability	Oceanographic devices, sensors and samplers	×	×	×	×	×	×	×	×
SSN		2011	Generic sensor	SSO	Semantic interoperability	Properties, measurement capabilities and observations	×	√	×	×	×	×	×	×
SemSorGrid4ENV		2011	environment monitoring system	N/a	semantic-based sensor network applications for environmental management	Common observation data model	×	×	×	×	×	×	×	×
SECURE		2011	environment sensors	SSO		Data from Environmental observation	×	×	×	×	×	×	×	×
SWROAO		2011	aircraft, ground and spacecraft sensors	N/A	Atmospheric monitoring	Satellite orbit, remote sensing and ground observation platform	×	×	×	×	×	○	○	×
SCO		2012	Generic sensor	SSO	Weather monitoring	Component, the Service and the Context module	×	○	×	×	×	○	○	×
IoT.est		2012	IoT sensors	SSO	IoT observation management	Resources, observations and measurement systems	×	○	×	×	×	×	×	×
Semantic Perception		2012	Environment monitoring machines	SSO	Environment perception	Observation and environmental knowledge	×	×	×	×	×	×	×	×
StarFL		2014	Generic sensor	N/A	Sensor discovery	Measurement capability	×	○	×	×	×	×	×	×
New SSN		2017	Generic sensor	SOSA	Broadening the Sensor application	Sensor, observation, sampler and actuator	×	√	×	×	×	×	×	×

Notes: √ Supported; ○ Partially Supported; × Unsupported.

**Table 2:** Comparison between the SOCA and SSN ontologies.

	Ontologies	System Capabilities in SSN	Observation Capabilities in SOCA
Features			
Measurement		*ssn:accuracy, ssn:drift* *ssn:resolution* *ssn:responseTime* *ssn:selectiveity* *ssn:frequency, ssn:measurementRange* *ssn:precision* *ssn:actuationRange* *ssn:latency* *ssn:repeatability*	*oca:FOV, oca:SwathRange, oca:GroundResolution, oca:BandWidth, oca:BandsWidthRange, oca:PolarizationFrequency, oca:RangeResolution, oca:NadirResolution, oca:RadiationResolution, oca:GeolocationAccuracy, oca:AngleAccuracy, oca:DistanceAccuracy, oca:RadiometricAccuracy, oca:AzimuthResolution, oca:IFOV, oca:SwingAngle, oca:RevisitCycle*
Category-Observation		-	*oca:BandsCategory, oca:BeamMode, oca:BandType, oca:PolarizationBand, oca:ObservedParameter, oca:PotentialApplication, oca:ThemeType*
Count-Observation		-	*oca:BandsNumber*
Truth-Observation		-	*oca:IsSwing*
Temporal-Observation		-	*time:Equals, time:Finishes, time:During, time:Starts, time:Overlaps, time:Meets and time:Before*
Geometry-Observation		-	*oca:SpaceEquals, oca:SpaceDisjoint, oca:SpaceIntersects, oca:SpaceTouches, oca:SpaceCrosses, oca:SpaceOverlaps, oca:SpaceWithin and oca:SpaceContains*
Complex-Observation		-	*oca:OpticalSpectralFeature, oca:RadarSpectralFeature, oca:RSImageData* *oca:SpaceCorrelation, oca:TimeCorrelation*

**Table 3:** Self-evaluation of SOCA ontology based on OntoQA metrics.

Evaluation Metrics and Their Precise Definition	Evaluation Process and Result	Evaluation Description
**Property Richness (PR)** Reflecting that more properties are defined, the more knowledge the ontology conveys.	PR = P/C = 81/75 = 1.08	Every class contains 1.08 properties, which means our ontology can convey a lot of domain knowledge to a certain extent (PR = 1.08).
**Inheritance Richness (IR)** Distinguishing a horizontal ontology from a vertical ontology or an ontology with different levels of specialization. A high IR means that ontology represents a wide range of general knowledge.	IR = SC/C = 37/75 = 0.49	It means that the horizontal ontology represents the knowledge in detail relatively.
**Relationship Richness (RR)** Reflecting the diversity of relations and placement of relations in the ontology. RR close to zero would indicate that most of the relationships are class-subclass relationships. RR close to one would indicate that most of the relationships are other than class-subclass.	RR = OP/(SC+OP) = 41/(37 + 41) = 0.53	The richness of ontology relationships is 0.53, which means our ontology has the characteristics of a diversity of relations.
**Average Population (AP)** An indication of the number of instances compared to the number of classes. A high AP means that the instances extracted into the knowledgebase might be sufficient to represent all of the knowledge.	AP = I/C = 200/75 = 2.67	This means that the implementation of instantiation is relative sufficient in the process of forming knowledge base.
**Readability (Rd)** Indicating the existence of human readable descriptions in the ontology, such as comments and labels. A higher Rd, the more the availability of human-readable information.	Rd = Number of rdfs: comment + Number off rdfs: label = 166 + 166 = 332	This metric can be a good indication for users to query, understand and share the ontology.

## Abbreviations

There are many abbreviations used within this thesis. The list below highlights some abbreviations, but it is not exhaustive.

A3ME	Agent-based Middleware Approach for Mixed Mode Environments
CEOS	Committee on Earth Observation Satellites
CESN	Coast Environment Sensor Network
CRESDA	China Centre for Resources Satellite Data and Application
CSIRO	Commonwealth Scientific and Industrial Research Organization
CSW	Catalogue Service for the Web
CWRC	Changjiang Water Resources Commission
DynamicOC	Dynamic Observation Capability
EO	Earth Observation
MMI	Marine Metadata Interoperability
IoT.est	Environment for Service Creation and Testing in the Internet of Things
OGC	Open Geospatial Consortium
O&M	Observations and Measurements
OSCART	Observing Systems Capability Analysis and Review Tool
RDF	Resource Description Framework
SANY	Sensors Anywhere
SCO	Semantic Component Ontology
SECURE	Semantics Empowered Rescue Environment
SemSorGrid4Env	Semantic Sensor Grids for Environmental Application
SensorML	Sensor Model Language
SIR	Sensor Instance Registry
SOCA	Sensor Observation Capability Association
SOSA	Sensor, Observation, Sample, and Actuator
SSN	Semantic Sensor Network
SSO	Stimulus-Sensor-Observation
StarFL	Starfish Fungus Language
StaticOC	Static Observation Capability
TSOC	Task-Sensor-Observation Capability
SWAMO	Sensor Web for Autonomous Mission Operations
SWE	Sensor Web Enablement
SWROAO	Sensor Web Resource Ontology for Atmospheric Observation
WMO	World Meteorological Organization
WWW	World Wide Web
